# A novel optimization algorithm for enabling dynamically collimated proton arc therapy

**DOI:** 10.1038/s41598-022-25774-2

**Published:** 2022-12-16

**Authors:** Blake R. Smith, Ryan T. Flynn, Daniel E. Hyer

**Affiliations:** Department of Radiation Oncology, University of Iowa, Iowa City, Iowa 52242 USA

**Keywords:** Radiotherapy, CNS cancer, Applied mathematics

## Abstract

The advent of energy-specific collimation in pencil beam scanning (PBS) proton therapy has led to an improved lateral dose conformity for a variety of treatment sites, resulting in better healthy tissue sparing. Arc PBS delivery has also been proposed to enhance high-dose conformity about the intended target, reduce skin toxicity, and improve plan robustness. The goal of this work was to determine if the combination of proton arc and energy-specific collimation can generate better dose distributions as a logical next step to maximize the dosimetric advantages of proton therapy. Plans were optimized using a novel DyNamically collimated proton Arc (DNA) genetic optimization algorithm that was designed specifically for the application of proton arc therapy. A treatment planning comparison study was performed by generating an uncollimated two-field intensity modulated proton therapy and partial arc treatments and then replanning these treatments using energy-specific collimation as delivered by a dynamic collimation system, which is a novel collimation technology for PBS. As such, we refer to this novel treatment paradigm as Dynamically Collimated Proton Arc Therapy (DC-PAT). Arc deliveries achieved a superior target conformity and improved organ at risk (OAR) sparing relative to their two-field counterparts at the cost of an increase to the low-dose, high-volume region of the healthy brain. The incorporation of DC-PAT using the DNA optimizer was shown to further improve the tumor dose conformity. When compared to the uncollimated proton arc treatments, the mean dose to the 10mm of surrounding healthy tissue was reduced by 11.4% with the addition of collimation without meaningfully affecting the maximum skin dose (less than 1% change) relative to a multi-field treatment. In this case study, DC-PAT could better spare specific OARs while maintaining better target coverage compared to uncollimated proton arc treatments. While this work presents a proof-of-concept integration of two emerging technologies, the results are promising and suggest that the addition of these two techniques can lead to superior treatment plans warranting further development.

## Introduction

The application of a rotational arc to deliver a pencil beam scanning (PBS) proton therapy treatment has recently been a growing area of interest in the field of radiotherapy. The concept was initially proposed for passive scattering proton therapy as a promising alternative for rotational electron therapy^1^. This idea was driven by the dosimetric qualities of protons, which exhibit a finite range and reduced range straggling as compared to electrons. Treatment planning studies had suggested that the integral lung dose resulting from a chest wall treatment could be reduced by half when delivered with a rotational passive scattering proton therapy treatment^1,2^. Furthermore, the uncertainties associated with protons, namely range uncertainty, can be reduced when delivered across a rotational arc^2^. Much of these potential benefits remained only theoretical until the advent of pencil beam scanning and accessible isocentric proton gantries.

Rotational proton therapy pushes the limits of practicality, even with the modern features of PBS, full 360$^\circ$ gantries, and fast energy layer switching. Traditional single-field uniform dose (SFUD) treatments achieve the desired dose distribution throughout the target by superimposing the dose contributions from multiple energy layers delivered at the same beam angle. Intensity modulated proton therapy (IMPT) is similar in that each beam angle has access to the full array of available beam energies to cover the extent of the target, albeit with the intent to not necessarily deliver a homogenous dose distribution from each beam angle. Proton arc therapy is complicated by the fact that the gantry rotation and beam energy are compounded: the delivered arc must be discretized in angular segments, each delivering only a single energy that will vary as a function of gantry angle. This dynamic creates a challenge to plan and optimize a treatment and has been the subject of many recent investigations.

Algorithm- and device-based solutions have been proposed to help facilitate proton arc treatments. The flexibility of PBS has lent itself well to algorithmic approaches to deliver a treatment using rotational arcs. Delivery-efficient PBS proton arc therapy algorithms have adapted control-point resampling, energy layer redistribution, and filtering to distribute a subset of energies among discretized arc segments using gradient descent methods^3,4^. The results from the proposed delivery technique show promising results, up to an 8% reduction in the integral dose and reduced skin dose by as much as 60% in comparison to two-field IMPT. A recent study has demonstrated using patient-specific quality assurance measurements that proton arc treatments are possible with state-of-the-art proton gantries^5^. Further developments in optimization have been able to improve the treatment efficiency by prioritizing energy transitions that are more efficient given the hysteresis of the scanning system^6^.

External collimation has been shown as an effective technique to improve dose conformity for low-energy (less than approximately 160MeV) pencil beam scanning (PBS) proton therapy treatments, and it is expected to play an increasing role in the treatment of brain and head and neck cancers^7^. Low-energy beams have larger lateral spot sizes that increase the lateral penumbra, especially for the cases where external range shifters are necessary to treat superficial targets. An external collimator can sharpen the lateral penumbra of the spot towards the healthy tissue thereby delivering a treatment with a plan quality of an effectively small spot size. The dynamic collimation system (DCS) is an emerging technology capable of mimicking any aperture shape through the sequenced motion of collimation trimmers^8,9^. Sequencing of the trimmers can be altered uniquely for each energy layer to provide energy-specific collimation that conforms to the lateral shape of the target for all energies delivered^10^.

Since its initial proposal, the DCS has progressed from a conceptual idea to a working mechanical prototype. Initial treatment planning studies have demonstrated a significant improvement in healthy tissue sparing and plan conformity relative to both aperture-collimated and un-collimated SFUD and IMPT treatment deliveries^11–14^. Specialized optimization algorithms have also been developed to maximize the achievable tumor dose conformity and determine the most efficient sequence pattern of the trimmers to deliver the treatment plan with minimal impact on the overall plan quality^10,15^. While not yet a clinical product, there have been advancements in modeling the primary and secondary radiations resulting from the DCS using Monte Carlo methods^16–18^ and developing a suitable calculation model with a commercial treatment planning system vendor^7^.

Combining energy-specific collimation with rotational arc therapy appears to be a natural step to further improve tumor dose conformity and healthy tissue sparing for PBS. However, it is not immediately clear whether the addition of collimation will be beneficial depending on the energy layer selection and departure from a classical per-field delivery approach that has driven the design and functionality of energy-specific collimators in PBS. Specifically, the extensive multiple Coulomb scattering experienced by protons traversing through tissue causes a lateral broadening of a proton beamlet, counteracting the effects of upstream collimation. Generally speaking, external beam collimation has a negligible effect at the Bragg peak of a proton beam once its energy surpasses 160 MeV^19^. This is important to consider for arc treatments as it could impact where collimation or beam energies should be prioritized, and it may be the case that collimation will not be beneficial if a significant portion of an arc requires high-energy protons to reach the target. It is also evident that the design and optimization of such an approach will be a complex task given the degrees of freedom that must be considered as they reflect the nature of the delivery. The purpose of this work was three fold: (a) to propose a new treatment paradigm in PBS, (2) apply a novel treatment optimization strategy to create arc-based PBS proton therapy plans, and (3) to show, via proof-of-principle, the dosimetric potential of dynamically collimated proton arc therapy, referred from hereon as DC-PAT.

## Methods and materials

### Optimization algorithm

A genetic optimization sequence was designed and integrated into a research-based treatment planning system to create DC-PAT plans. Genetic algorithms are part of a class of evolutionary algorithms, which are stochastic problem solvers that operate based on the biological process of natural selection. A genetic algorithm design is often used to generate a subset of superior solutions when the problem includes a complex solution space with many covarying components^20^.

**Figure 1:**
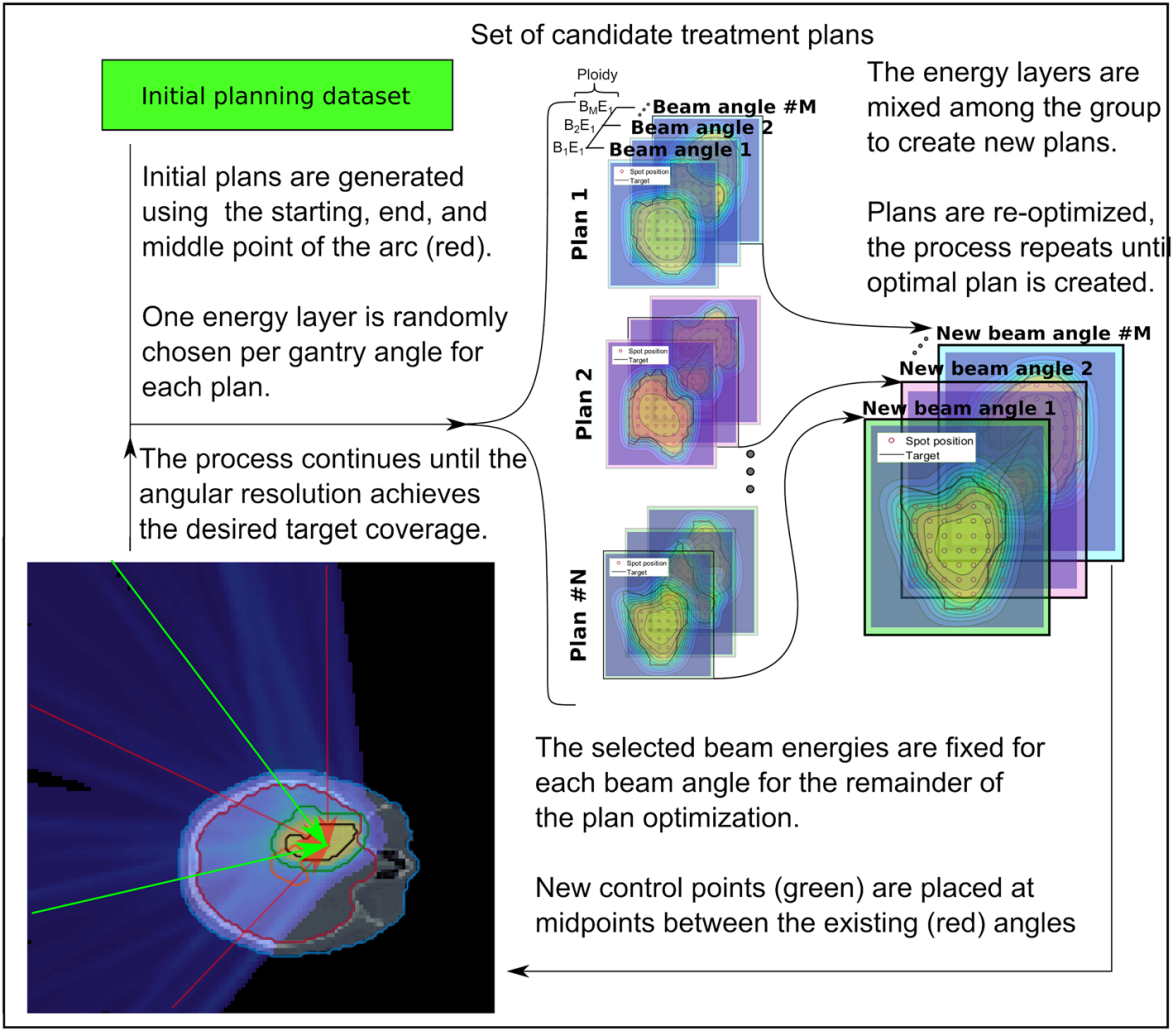
Algorithm flow chart of the arc-based treatment planning strategy used to optimize the DC-PAT treatments. The ploidy is defined by the number of energy layers per beam angle that are considered. A haploid system is presented above were each gantry angle has only a single energy assigned to it.

The general workflow of the algorithm is illustrated in Fig. 1. An initial population of candidate solutions is generated and organized based on the properties specific to the problem, referred to as its genotypes. Unlike conventional beamlet optimization techniques where a candidate plan is solely expressed as a set of individual beamlet weights, each member is uniquely represented as a haploid chromosomic band where each nucleotide dictates the energy layer that is delivered in the treatment sequence as a function of gantry angle. Several members are generated to form a population, and the population evolves over multiple iterations, or generations. Beamlet weights for each member are continuously optimized as a function of their assigned energy layer and gantry angle. After each generation, the members of the population are ranked using a DVH-based objective function, *F*, shown in Eq. 1 that compares the current dose distribution, $d_{i}$, against the constraints of the target, $\xi$, and nearby organs at risk, *k*^21^. The full set of variables are listed in Table 1.

1 $$\begin{aligned} \begin{aligned} F= \sum _{k=1}^{K} \left( \frac{1}{T_{k}} \sum _{i \in \tau _{k}} \left[ \beta _{k}^{+} c_{(0,\infty )}^2 (d_{i}-d_{i,k}^{+}) + \beta _{k}^{-} c_{(-\infty , 0)}^2 (d_{i}-d_{i,k}^{-})  \right. \right. \\ \left. \left. + \beta _{k}^{V_{k}^{+}} c^2_{(0,\Delta D_{k}^{V_{k}^{+}})} (d_{i}-d_{i,k}^{V_{k}^{+}}) + \beta _{k}^{V_{k}^{-}} c^2_{(\Delta D_{k}^{V_{k}^{-}},0)} (d_{i}-d_{i,k}^{V_{k}^{-}}) \right] \right) , \end{aligned} \end{aligned}$$

where,

2 $$\begin{aligned} c_{(a,b)}(x)= {\left\{ \begin{array}{ll} x &{} \text { if } a\le x \le b\\ 0 &{} \text { else }\\ \end{array}\right. }. \end{aligned}$$

**Table 1:** Definitions of variables used in Eq. 1.

Variable	Description
$\tau _{k}, T_{k}$	Subset voxel indices and number of voxels, respectively, that are inside tissue *k* with $k \equiv \xi$ denoting the target
$d_{i,k}^{\pm }, \beta _{k}^{\pm }$	Dose threshold for voxel *i* and penalty weight, respectively, for overdose(+) and underdose(-) penalties for tissue *k*
$d_{i,k}^{V\pm }, V_{k}^{\pm }, \beta _{k}^{V_{k}^{\pm }}$	Dose threshold and penalty weight, respectively, for dose-volume overdose(+) and underdose(-) penalties for tissue *k*
$\Delta D_{k}^{V_{k}^{\pm }}$	Difference between the dose planned and desired to $V_{k}^{\pm }$ in the cumulative dose-volume histogram for tissue *k*

The highest-ranked member, denoted as the alpha, is cloned and is automatically included within the next generation. The remaining members undergo probabilistic mating where their offspring are the result of randomly selected energy layers between their parent and a partner member. The partner is randomly chosen where the highest-performing constituents have a proportionally higher probability of mating thereby promoting the best organization of energy layers that are distributed over the arc. The lowest-performing members, referred to as the runts, are killed off and replaced with new members who have freshly generated nucleotides to promote genetic diversity within the genetic system. This optimization process is illustrated for a single sequence in Fig. 1.

The genetic complexity of the system increases over time and is organized in evolutionary stages. The first stage contains only constituent members which are made up of three beam angles that consist of the starting, stopping, and middle gantry angles over the intended arc length. As part of the process shown in Fig. 1, the resultant alpha optimized over multiple generations from the first evolutionary stage is carried over to the next stage where additional beam angles are placed between the existing gantry angles of the previous stage. The energy layers at each of the gantry angles that were determined from the previous stage are fixed and can not be changed for the remainder of the optimization. Beam energies at a specified gantry angle are only optimized at their respective evolutionary stage. While beam energies are fixed from earlier stages, their resultant dose distributions can change at any course of the optimization process as the beamlet weights across all energy layers are continuously optimized at the start of every generation for each member.

The addition of collimation was then applied to the solution from the genetic optimizer, referred to as the DyNamically Collimated Arc (DNA) optimizer. Beamlets were collimated on an individual basis to maximize the tumor dose conformity of the entire treatment plan. Arc treatment plans were organized with a single energy layer per incremental gantry angle to simplify the genetic encoding of a member as a haploid, rather than as a polyploid. The spacing between subsequent gantry angles were iteratively reduced until a satisfactory solution could be achieved. Arc-based treatment plans were compared against their uncollimated two-field IMPT counterparts.

### Treatment planning studies

**Figure 2:**
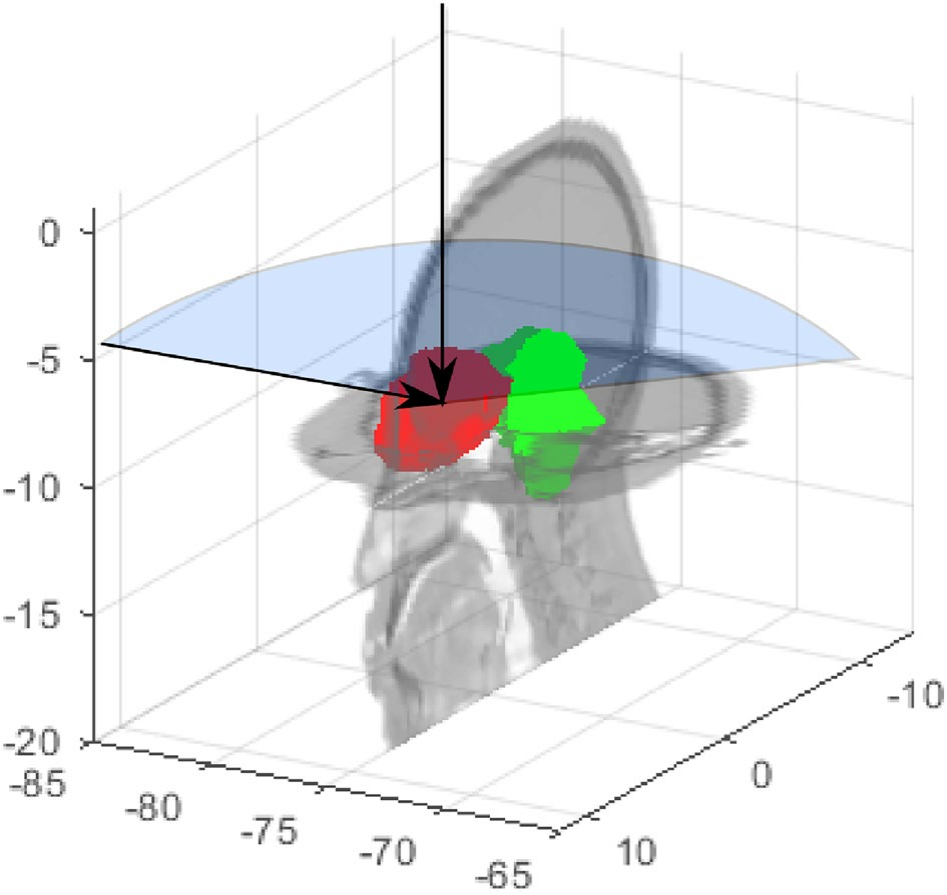
Treatment planning geometry of the patient, target (red), and brainstem OAR (green). Beam directions are shown for the multi-field IMPT treatments as represented by solid arrows and the partial arc bounded by the blue sector. Plot coordinates are in units of centimeters.

A set of proton arc treatment plans was created for an intracranial treatment of a chordoma target illustrated in Fig. 2. All treatments consisted of a single axial arc starting from the lateral right of the patient and wrapping posteriorly around the patient. Spots were delivered in a uniform grid with a lateral spot spacing of 4mm and energy layers per gantry angle were spaced uniformly in 5mm water-equivalent thickness intervals. Uncollimated proton dose distributions were calculated using Hong’s algorithim^22^ modeled after the IBA UN system at Northwestern. Beamlets collimated by the DCS were calculated using the asymmetric beamlet algorithm by Gelover et al, which is a regressed analytical model from experimentally benchmarked Monte Carlo simulations^23^.

Multiple treatment plans were created to evaluate the impact of energy layer placement and collimation on the overall plan quality. A conventional two-field treatment plan was first optimized with and without energy-specific for the right lateral and an apex beams. The anonymized CT planning dataset used in this work was from an institutional review board (IRB) approved study at the University of Pennsylvania and shared through an institution-approved agreement following the approved methods and protocols. As part of the IRB-approved study, informed consent was obtained from all subjects. The delivery reflects a clinical standard of care, including beam angles, lateral and distal spot spacing, which were taken from the original plan to closely resemble the nominal planned dose distribution^11^. A series of uncollimated and DC-PAT treatment plans were then optimized over a 140$^\circ$ arc and weighted to achieve a representative $D_{{95}{\%}}$ coverage of 50Gy to the intended target. Each arc began as a set of three evenly spaced control points 70$^\circ$ apart that was evolved seven times, resulting in 129 control points treated as discretized 1.1$^\circ$ sub arcs.

Two special case studies were performed to investigate the solution stability and organ at risk (OAR) avoidance capabilities. The first case entailed an alternative optimization approach that mimicked a scenario where the maximum dose to the brain stem had to be minimized, such as a clinical case of a patient re-treatment. Uncollimated- and DC-PAT treatments were planned to achieve as much (a) nearby healthy tissue sparing while maintaining a minimum target coverage of the 50Gy prescription to 95% of the target volume and (b) by limiting the maximum dose on the brainstem. For the latter case, a highly-weighted maximum dose goal of 10Gy to 2% of the brain stem was placed on the brainstem. It is important to note that only soft constraints are available in our in-house TPS and some small deviations are expected, especially since conflicting goals are set to achieve target coverage. A separate study focused on the stability of the genetic optimization process using a series of ten genetic optimizations that were repeated using different random number generator seeds. The distribution of energies optimized for each gantry control point was determined as well as the variance of the resulting coverage across the target and the surrounding regions of interest.

For the specific patient studied, the dose to the target, brainstem, healthy brain, 0.5cm skin thickness from the body of the patient, and the immediately surrounding 10mm of healthy tissue were quantified using dose-volume histograms (DVH) plots. Specific metrics were calculated from the DVHs including the target coverage defined as the dose to 95% of the target volume ($D_{{95}{\%}}$), regional high-dose to 2% of an ROI’s volume ($D_{{2}{\%}}$), and the mean dose to an ROI ($D_{\text {mean}}$). Integral dose was also calculated for each plan based on the dose-volume summation to the healthy tissue outside of the 10mm of tissue surrounding the target while within the skin.

## Results

### Treatment planning comparison

**Figure 3:**
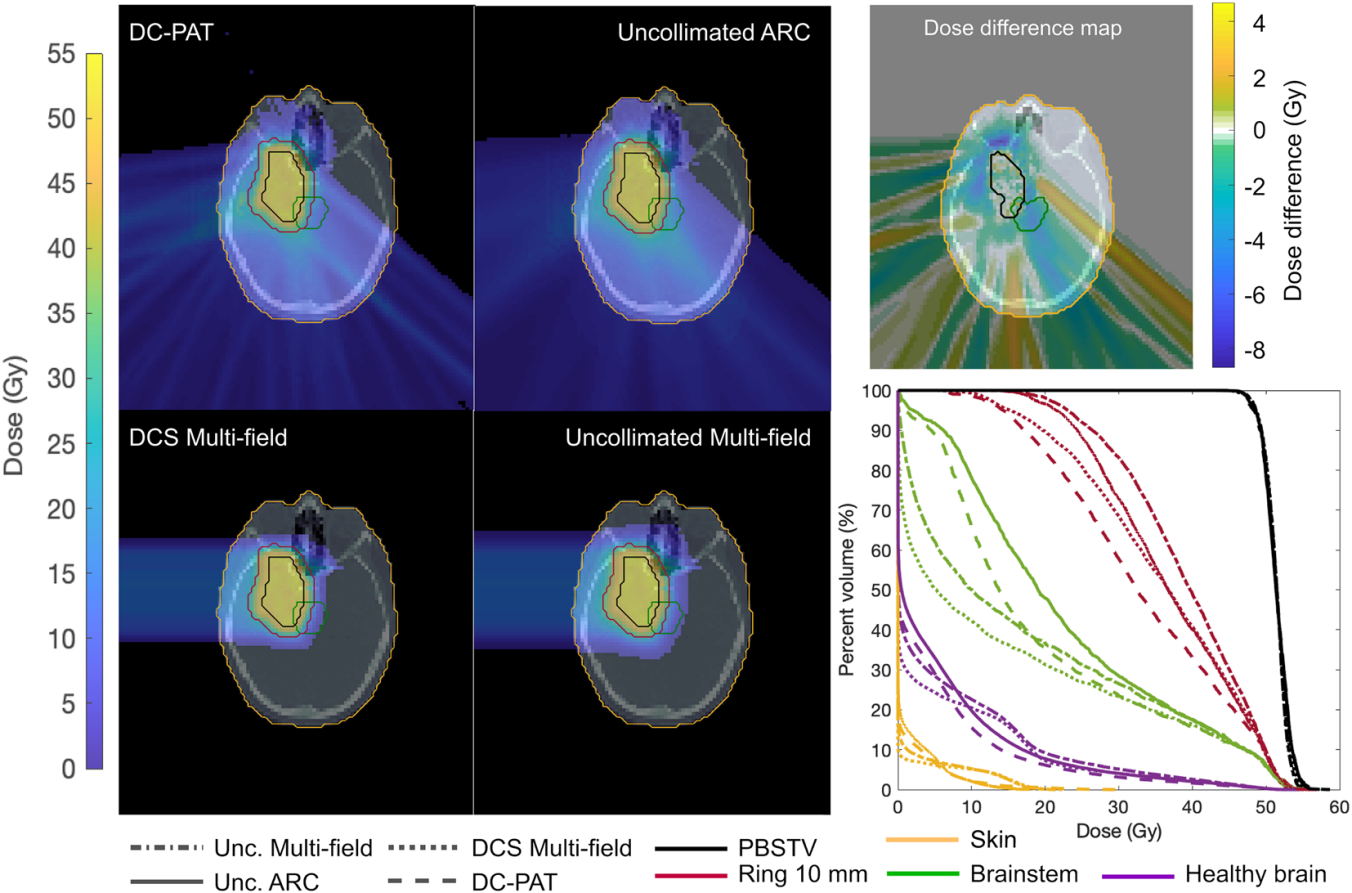
Dose distribution and dose-volume histogram (DVH) from the multi-field and proton arc treatment plans that were delivered with (DCS) and without (Unc) energy-specific collimation. Arc treatment plans spanned a 140$^\circ$ gantry angle are subdivided into 129 control points where each control point either consisted of a single energy layer (Unc ARC and DC-PAT). A separate plot is presented showing the difference between these two dose distributions. The coverage is shown for the pencil beam scanning target volume (PBSTV), 10mm ring of healthy tissue surrounding the PBSTV (Ring 10mm), brainstem, and the healthy brain.

Treatment plans were optimized to nominal coverage of 50Gy, which was the normalization point of the dose to 95% volume when comparing dose volume histograms. There are four treatment planning DVH plots shown in Fig. 3 that span multiple treatment planning strategies to maximize plan conformity including multi-field and arc delivery approaches. Rotational proton arc deliveries consisted of an arc that spanned 140$^\circ$ within the transverse plane of the patient such as would be delivered using a full 360$^\circ$ PBS-enabled gantry. Each arc was subdivided into 129 control points that were separated by 1.1$^\circ$. Each control point consisted of only a single energy layer. Optimization goals were similar among each of the plans and were placed on the target coverage, sparing of the 10mm rind of tissue that encompassed the pencil beam scanning target volume (PBSTV). An additional penalty minimizing the maximum dose to the volume of healthy brain was used for the proton arc treatments.

Progression to an arc delivery from a static multi-field delivery improved the target conformity and healthy tissue sparing. As summarized in Table 2, the dose to 50% of the volume ($D_{50}$) of the 10mm ring was 11.2% lower when planned with an arc versus a multi-field delivery for the uncollimated deliveries. Improvements over multi-field treatments were also observed among large critical structures, specifically the integral dose to the healthy brain tissue outside the 10mm ring, which was 1.1% lower when delivered using a rotational arc whereas the maximum skin dose was reduced by 34.6%. The addition of collimation provided an additional 11.4% reduction to the mean dose of surrounding tissue with a minimal change, less than ${1}{\%}$, to the maximum skin dose. Since the arc delivery partially traversed the brain stem, the corresponding $D_{50}$ was nearly doubled compared to the multi-field approach. The maximum brainstem dose and target coverage were unchanged, less than 1.5%, between an arc and a multi-field treatment delivery.

**Table 2:** Dose-volume histogram results of the treatment planning studies by delivery technique.

Plan	PBSTV	10mm Ring		Skin	Brain		Brainstem	
Name	$D_{2}$(Gy)	$D_{90}$(Gy)	$D_{50}$(Gy)	$D_{2}$(Gy)	Integral (Gy cm$^{3}$)	$D_{2}$ (Gy)	$D_{50}$ (Gy)	$D_{2}$ (Gy)
Unc ARC	56.5	25.1	37.8	10.4	6.40$\times$10$^{3}$	41.6	19.6	53.7
DC-PAT	56.8	17.8	33.5	10.5	5.43$\times$10$^{3}$	38.5	14.2	54.4
Unc multi-field	55.6	30.2	40.8	16.0	6.47$\times$10$^{3}$	43.7	10.3	53.3
DCS multi-field	56.0	20.2	37.7	16.4	5.52$\times$10$^{3}$	42.2	5.4	53.4

### OAR avoidance

**Figure 4:**
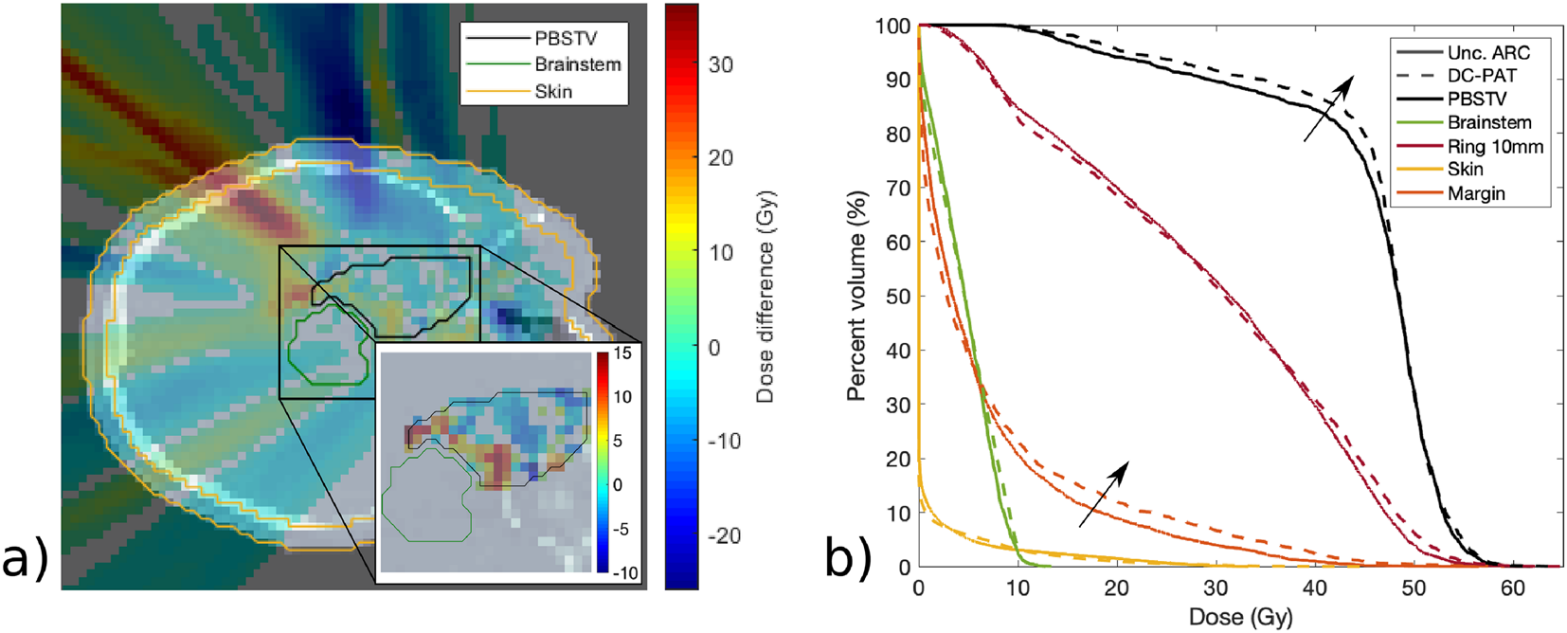
(**a**) Dose difference map between the DC-PAT and uncollimated arc PBS treatment plans treating a reduced target volume in order to spare the brainstem. A zoomed-in profile of the dose difference is shown for voxels within the pencil beam scanning target volume (PBSTV). A new structure referred to as the Margin was included within the DVH and is the portion of the PTV that is within 5mm of the brain stem contour. (**b**) DVH of the nearby structures is shown right. Each plan was optimized to achieve as much target coverage as possible while maintaining a nearly equivalent brainstem DVH.

Using the optimized treatment plans from the first case, a maximum dose threshold of 10Gy was placed on the brainstem and was weighted so that it constrained the optimization to solutions that satisfied this criteria while achieving as much target coverage as possible. Additional constraints were also used to limit the $D_{{2}{\%}}$ within the PTV to within 112% and a $D{{50}{\%}}$ of at least 96% of prescription. Figure 4 shows the resulting dose profiles and composite DVH from the uncollimated and DCS collimated treatments for this scenario. The delivery intent of the treatment plan was 50Gy to 95% of the target volume. The achieved target coverages for each plan are listed in Table 3.

**Table 3:** DVH coverage metrics following a treatment plan with and without DCS collimation that prioritized a maximum brain stem dose goal.

Plan	PBSTV			10mm Ring	Skin	Brain stem
Name	$D_{2}$(Gy)	$D_{90}$(Gy)	$V_{{90}{\%}}$(%)	$D_{50}$(Gy)	$D_{2}$(Gy)	$D_{2}$(Gy)
Unc. ARC	56.0	29.0	74.6	31.3	16.7	10.1
DC-PAT	56.4	34.1	79.5	30.8	13.9	10.2

It is interesting to note the dose differences presented in Fig. 4. To achieve as much target coverage as possible, both the uncollimated and DCS-collimated treatment plans place significant weight among a few beam angles. The large region of lateral tissue towards the right eye receives a higher dose from the uncollimated plan, likely owing to the collimating capabilities of the DCS that are apparent for lower beam energies. Most notably, the utility of energy-specific collimation can be seen where the PBSTV abuts the brainstem where the sharper penumbra afforded by the DCS allows an increase in the PBSTV dose coverage.

### Genetic optimization convergence

**Figure 5:**
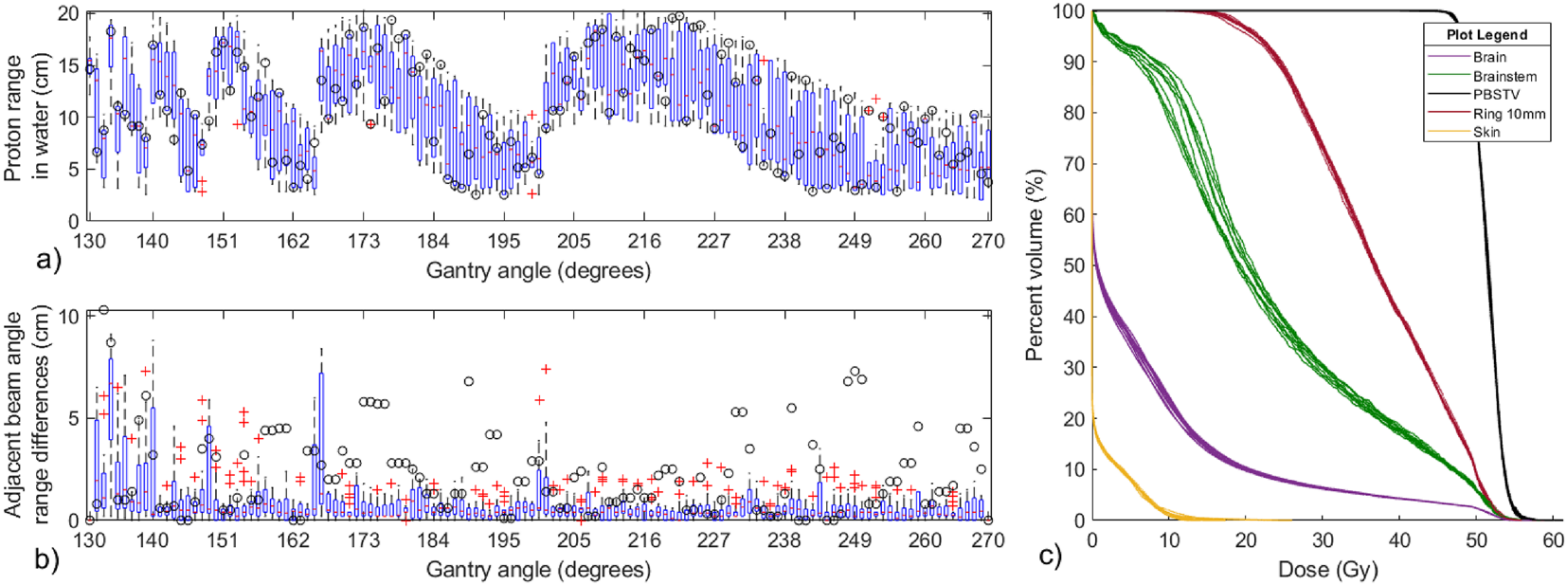
(**a**) Histogram of proton range layers for each gantry angle control point among 10 independently optimized treatment plans. The proton beam range for each gantry angle control point is plotted $\left( \circ \right)$ for a single planning case. (**b**) The distribution of adjacent energy layers among the 10 treatment plans is plotted below for each gantry angle (see text for details). The minimum proton range difference between adjacent beam angles is shown for a single case, $\left( \circ \right)$ . (**c**) An overlay of 10 DVH profiles resulting from the dose distribution optimized from ten independent genetic proton arc optimizations.

Figure 5 shows the overlay of the ten DVH profiles resulting from each independent optimization. Minimal variation was observed in the DVH profiles. The target $D_{{95}{\%}}$ and $D_{{2}{\%}}$ coverage remained within 0.16% and 0.31%, respectively, of the prescription for all optimization trials. Less than a 0.4% change was observed in the $D_{{50}{\%}}$ for the surrounding 10mm of healthy tissue and the max, $D_{{2}{\%}}$, of the skin range between 8.7Gy and 9.9Gy for all trials. Some variability was observed for the brainstem; while the maximum dose remained nearly unchanged, the $D_{{50}{\%}}$ spread was 3.5% and increased for lower-dose, high volume regions.

There was a large variation in the selected energy delivered at a particular control point among the optimized plans as shown in Fig. 5. Effective range in water was observed to vary by as much as 10cm for the same control point between independently optimized plans while still producing a very similar dose distribution and DVH as shown in Fig. 5. For the specific patient studied, large variations in the planned energy layer were consistently observed for gantry angles between 200$^\circ$ and 270$^\circ$. The similarity of treatment plans was further investigated by evaluating the sequence of energy layers between plans across adjacent control points. At each control point, the difference between one plan’s beam range to the previous and next gantry angle control points of the nine other treatment plans was computed, where the nearest beam range from another treatment plan was recorded. This was repeated for all 10 treatment plans at the same control point, generating a distribution of nearest beam range differences between adjacent gantry control points among different treatment plans, which is shown in the bottom box plot of Fig. 5.

## Discussion

This work is the first demonstration of DC-PAT. This work demonstrates that combining energy-specific collimation with PBS in an arc-style delivery can provide further dosimetric improvement including increased tumor dose conformity than if either of these two technologies were utilized separately. The addition of collimation has been shown to provide increased healthy tissue sparing for a variety of treatment sites, most notably low-energy treatments with large spot sizes or if an external range-shifting device is necessary to achieve the desired proximal coverage. One potential disadvantage of collimated PBS proton therapy is that the surface dose is elevated from the low-energy scattered protons from the collimators. Consequently the maximum skin dose, which is already higher in general for proton therapy treatments, is further increased. Intuitively, proton arc deliveries are an attractive option for collimated deliveries as the entire dose distribution is delivered over a larger skin area.

The DNA-optimization approach offers a unique perspective into plan optimization behavior for proton treatments. Proton treatments are presented with a unique optimization challenge of determining an optimal distribution of beam energies relative to the gantry position. While deterministic optimizers have been used to solve this problem previously, it is not immediately clear from these works how sensitive this relationship is to deviations to the beam energy versus gantry angle. The results from this work suggest that there is a large distribution of potential beam energy and gantry angle arrangements that can achieve nearly the same dose distribution. While this is true, the results plotted in Fig. 5 suggest a lack of consistency among treatment plans. However, a deeper look reveals that the average change in proton range between adjacent energy layers is between 0cm and 5.82cm depending on the gantry angle among all 10 treatment plans. The distribution of proton range differences between the adjacent beam angles among the 10 optimized treatment plans is plotted in Fig. 5, showing that for most cases similar beam ranges are used within a small angular segment of one plan to another.

The flexibility afforded by DNA-optimization may be exploited to further restrict the solution space with stricter search criteria or alternative criteria such as time-efficient delivery sequencing. Alternatively, a wide distribution of candidate energies for each gantry angle may also warrant further investigation and development to deliver proton therapy treatments with a poly-energetic spectrum of variable spread-out Bragg peak width. While there are technological obstacles that hinder such a delivery, it would present an easier optimization problem with fewer degrees of freedom and potentially improve the plan quality within the arc delivery. Furthermore, LET-dependent models could be incorporated into the proton dose distribution to enhance planning optimization and energy selection techniques based on normal tissue toxicity rather than dose conformity following normal tissue radiosensitivity models, which have been parameterized as a function of LET^24^.

A direct comparison to previous works is difficult given the novel nature of this treatment paradigm, the differences in optimization functionality, and the differences in intended treatment sites. Furthermore, it should also be noted that the results from this work showcase idealized dose distributions. Given the novelty of the DCS, robust optimization features to account for range and setup uncertainty are not included in this study as these features have not been develop but are a current area of development as the first FDA-approved treatment planning system is being modified to accurately model a DCS with PBS^7^. However, similar works have reported improved integral dose, reduced maximum skin dose, and increased tumor dose conformity which is reflected in our results. As shown from the 10mm ring DVH plots in Fig. 3, the addition of collimation improved the conformity of the plan. The dose in the region immediately surrounding the PBSTV was reduced with the incorporation of collimation. However, this sparing effect did not present uniformly. As shown in Fig. 3, the majority of sparing around the PBSTV occurred cross-plane throughout the arc that defines the longitudinal extent of the target superiorly and inferiorly. A notable reduction to the healthy tissue dose was also observed towards the angular portion of the target that requires the least depth of penetration from the patient surface as well as at locations where the periphery of the target was positioned at the apex of intersecting, non-oblique scanning angles delivered during the arc. This presents as a *criss-crossed* dose difference map between the DC-PAT and uncollimated arc treatment plans.

Some additional optimization features could be devised to improve the stochastic methods employed in this work. From the presented results, it appears that a genetic-based design offers several advantages including a wider solution space sampling and independence from an assumed initial starting position. The presented algorithm also offers a convergent design that sequentially optimizes a subset of control points before increasing the resolution of the arc. In this regard, the optimizer functions in part as a heuristic search algorithm that solves the problem in portions. The unique sequence that describes each individual member is constructed from this iterative process. A large consequence of the genetic algorithm design will be the additional computational resources given that multiple treatment plans are continuously optimized. Using the genetic population parameters in this study, arc-style treatment plans required nearly 10 times longer than their multi-field counterparts to optimize on a single core. While a notable increase, this is not expected to be clinically prohibitive, especially given that multiple portions could be solved simultaneously with multiple energy layers potentially considered for each gantry angle by increasing the ploidy of the population that is optimized. The current scheme of limiting a single energy layer to a gantry angle makes the delivery more similar to representing a true arc, but further treatment design should also be considered that places beam spots along the curvature of the arc to better simulate the delivery. While not explicitly investigated in this work, it would seem reasonable to presume that the robustness and efficiency of DCS treatments would also improve relative to what has been estimated for static, two-field deliveries.

## Conclusions

Both an arc delivery and dynamic collimation can improve plan conformity relative to a contemporary two-field IMPT treatment. The use of a single arc was found to reduce the median healthy tissue dose immediately surrounding the PBSTV, which is consistent with the improvement reported by similar developments in proton arc delivery optimization. DC-PAT provided improved tumor dose conformity relative to the multi-field IMPT treatment while preserving the reduction afforded by an arc delivery to the high dose region of the healthy brain and skin.

## Data Availability

The data that support the findings of this study are available from the corresponding author upon reasonable request.
